# Shortcoming of ypStage staging system: Lack of differentiation for preoperative treatment

**DOI:** 10.1371/journal.pone.0318854

**Published:** 2025-03-13

**Authors:** Peizhun Du, Jinzhe Zhou, Pengcheng Liu, Guangjian Huang, Cheng'en Hu

**Affiliations:** 1 Gastrointestinal Surgery Center, General Surgery Department, Huashan Hospital, Fudan University, Shanghai, China; 2 Department of General Surgery, Tongji Hospital, Tongji University School of Medicine, Shanghai, China; Affiliated Hospital of Nanjing University of Chinese Medicine: Jiangsu Province Academy of Traditional Chinese Medicine, CHINA

## Abstract

The eighth edition of the AJCC staging manual initially proposed the ypTNM staging system, which was specifically designed to assess the staging and predict the prognosis of cancer patients undergoing preoperative treatment. Nevertheless, it remains unclear whether this staging system is an accurate predictor of outcomes for cancer patients undergoing different preoperative treatments. The clinical and pathological data of gastric cancer patients who received preoperative treatment and subsequent curved tented gastrostomy were obtained from the Surveillance, Epidemiology, and End Results database. A comparison of survival rates was conducted between patients with the same ypStage staging who received preoperative chemotherapy or chemoradiotherapy, using the Kaplan-Meier method. Additionally, a Cox regression analysis was performed to identify the factors influencing survival following preoperative treatment. A total of 202 patients were included in the study. The results demonstrated a statistically significant difference (p <  0.05) in survival between patients who received preoperative chemoradiotherapy and those who received preoperative chemotherapy in ypStage II or III patients.Cox regression analysis revealed that ypT, ypN and ypStage were associated with OS, but were not independent prognostic factors following gastrectomy. The survival of gastric cancer patients who are classified in the same ypStage stage but who receive disparate preoperative treatments is not analogous. The eighth edition staging system remains in need of further refinement to ensure accurate prediction of prognosis following diverse preoperative therapeutic regimens.

## Introduction

Gastric cancer (GC) is the fifth most common malignancy worldwide and the third most deadly [1]. Surgery remains the most common and effective treatment for localised GC. However, despite radical resection, survival rates for patients with advanced GC remain low, the 5-year overall survival (OS) is mostly less than 50% [2]. Some patients have even lost the opportunity for surgical resection at the time of diagnosis. To improve the resectability rate of GC patients and prolong the survival time of postoperative patients, Wilke *et al* proposed the strategy of preoperative radiotherapy or chemotherapy [3]. Research has shown that preoperative chemotherapy can improve patient tolerance, increase the rate of radical resection, reduce tumour metastasis, and ultimately prolong patient survival [4]. In addition to chemotherapy, preoperative radiotherapy has also been shown to achieve tumour downgrading. Preoperative treatment combined with surgery has become one of the options for the treatment of GC. With the increasing use of preoperative therapy, the prediction of survival after preoperative treatment for GC has also become a notable issue.

The American Joint Committee on Cancer (AJCC) TNM staging system has historically been employed to evaluate the severity of tumour and forecast its prognosis. This system is primarily based on the depth of tumour infiltration (T), lymph node metastasis (N) and distant metastasis (M). The extent of disease prior to surgical intervention is determined by clinical TNM (cTNM), while the extent of disease subsequent to surgical resection is determined by pathological TNM (pTNM). Patients with the same stage often exhibit comparable survival rates. It is possible that preoperative treatment may result in the death of some tumour cells and lead to tumour regression, which would reflect the tumour’s response to preoperative treatment. It is important to differentiate between patients who have undergone preoperative treatment and those who have not, with regard to their pathological staging.

The eighth edition of the AJCC manual was the first to describe the staging system for patients who receive preoperative therapy [5]. This represents a significant advance in precision therapy and provides a robust framework for patient evaluation following preoperative treatment. This system employs the terms ‘ypStage’ to quantify the actual degree of tumour at the time of surgery and predicts the postoperative survival of GC patients who received preoperative treatment. However, it is worth noting that there is more than one option for preoperative treatment including chemotherapy, radiotherapy, targeted therapy, immunotherapy and so on. It is pertinent to inquire whether the prognosis will be analogous for patients with an identical postoperative ypStage who have undergone disparate preoperative treatments (such as chemotherapy or chemoradiotherapy). It would be beneficial to ascertain whether it is necessary to specify ypTNM according to different preoperative treatments.

The Surveillance, Epidemiology, and End Results (SEER) database, established by the National Cancer Institute in 1973, is a leading and comprehensive resource among the major tumour databases in the United States [6]. Over the course of several decades, the database has amassed a substantial corpus of information pertaining to mortality, prevalence, incidence, and other empirical medical data on patients with tumours in a multitude of states and counties across the country.

In this study, we conducted an analysis of data pertaining to GC from the SEER database, with the objective of comparing the survival rates of GC patients who had received preoperative chemotherapy or chemoradiotherapy and had the same postoperative pathological stage. Analysis indicated it is inaccurate to rely solely on postoperative pathology to guide the prognosis of GC patients after preoperative therapy, as this approach ignores the potential influence of preoperative treatment plans.

## Methods

### Data source and study population

The patient clinical data were downloaded from the SEER 17 Regs Research database (Nov 2023 Sub (2000-2021)) using the SEER*Stat software on August 11, 2024. The SEER database was queried to extract data pertaining to patients with tumours located in the stomach (primary site: C16.0-16.9). The selection of variables was guided by the principle of comparing the impact of different preoperative treatment methods (chemotherapy or chemoradiotherapy) on patients’ postoperative prognosis. For each enrolled patient, the following data were collected: age, gender, race, T category, N category, M category, surgical status, radiotherapy status, radiotherapy/surgery sequence, chemotherapy status, chemotherapy/surgery sequence, regional lymph node resection, months of survival, and vital status.

### Inclusion/exclusion criteria

The following criteria had to be met for inclusion in the study: 1. A confirmed diagnosis of GC had to be provided. 2. Received preoperative chemotherapy or chemoradiotherapy. 3. Radical resection surgery was performed, and the T, N, and M category were determined based on the postoperative pathological outcomes. Patients who met the aforementioned criteria were excluded from the study: 1. Patients with more than one primary cancer. 2. Other neoplasms, including gastric stromal tumours, sarcomas, lymphomas and neuroendocrine tumours, were excluded.

### Statistical analysis

Categorical variables, such as gender, race, tumor size, grading, and TNM category, were reported as proportions and compared between groups using Chi-square tests. For continuous variables that did not adhere to a normal distribution, medians and interquartile ranges (IQR) were utilized, with comparisons made via the Mann–Whitney U test; for instance, age was analyzed in this manner. Survival analysis was conducted using the Kaplan–Meier method, and differences in survival rates between the chemotherapy group and the chemoradiotherapy group were assessed using the log-rank test. A two-tailed p-value less than 0.05 was considered statistically significant. Both univariate and multivariate analyses were performed using Cox proportional hazards models to calculate hazard ratios (HRs) and their 95% confidence intervals (CIs). Variables with a p-value less than 0.05 in the univariate analysis were included in the multivariate Cox regression model.

## Results

### Clinicopathological characteristics

**Fig 1:**
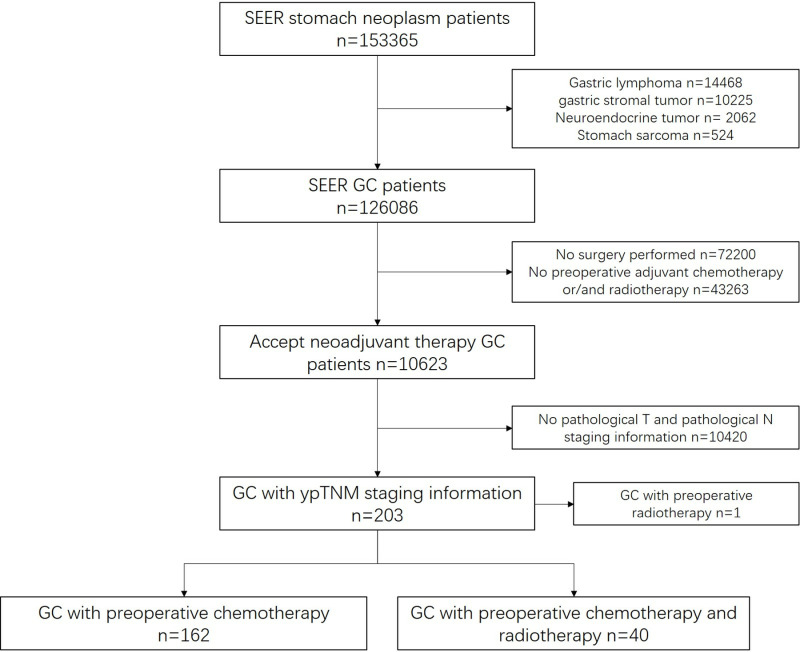
The flow chart of patient selection.

The data of 153,365 GC patients were downloaded from the SEER database for the purposes of this study. In accordance with the established inclusion and exclusion criteria, the analysis was conducted on a total of 203 patients from the period between 2016 and 2017. The 202 patients were divided into two groups: the preoperative chemotherapy group (n = 162) and the preoperative chemoradiotherapy group (n = 40). Fig 1 illustrated the process of case screening. The baseline characteristics of the patients were summarised in Table 1. The average age of the cases was 60 years, consisting of 118 males and 84 females, with 64.8% of the patients being White. YpStage has been reclassified according to the 8th edition of AJCC manual. Among the included patients, ypStage III patients accounted for the majority (n = 128).

**Table 1:** Baseline characteristics of GC patients received preoperative treatment and sequence gastrectomy.

Characteristic	Total patients n = 202	preoperative chemotherapy n = 162	preoperative chemorediontherapy n = 40	χ2	P value
Age					
Medians(IQR)		60.0 (50.7,70.0)	64.0 (54.2,71.7)		0.355
Sex					0.006
Male	118	87	31		
Female	84	75	9		
Race				3.83	0.050
White	131	98	33		
Black	26	24	2		
Other/unknown	45	40	5		
Size				1.12	0.569
<2 cm	11	8	3		
2-5 cm	58	46	12		
>5 cm	53	45	8		
Unknown^*^	80	63	17		
Grade				2.07	0.354
Well	3	2	1		
Moderately	25	18	7		
Poorly	152	126	26		
Unknown^*^	32	16	6		
ypT category				23.81	<0.001
T1	11	8	3		
T2	17	11	6		
T3	73	48	25		
T4	98	92	6		
ypN category				23.21	<0.001
N0	33	21	12		
N1	35	27	8		
N2	49	33	16		
N3	84	80	4		
Nx	1	1	0		
M category				1.28	0.257
M0	176	139	37		
M1	26	23	3		
ypStage				15.85	0.001
I	21	12	9		
II	27	17	10		
III	128	110	18		
IV	26	23	3		

* Not included in the analysis of differences between the two groups.

### Comparison of survival rates between chemotherapy and chemoradiotherapy groups

**Fig 2:**
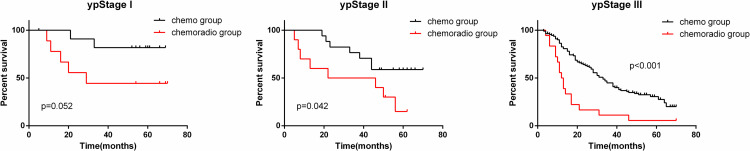
The overall survival rate was compared between patients who received preoperative chemotherapy and those who received chemoradiotherapy.

A comparison of the survival rates of patients in the same ypStage (I, II and III) between two groups was conducted. The results demonstrated that for patients in ypStage II (Fig 2B) or III (Fig 2C), the survival time of patients who received preoperative chemoradiotherapy was significantly shorter than that of patients who received only chemotherapy (p <  0.05). In patients with ypStage I (Fig 2A), the same trend was observed, but no statistically significant difference was identified (p =  0.052).

### Univariate and multivariate analysis

**Fig 3:**
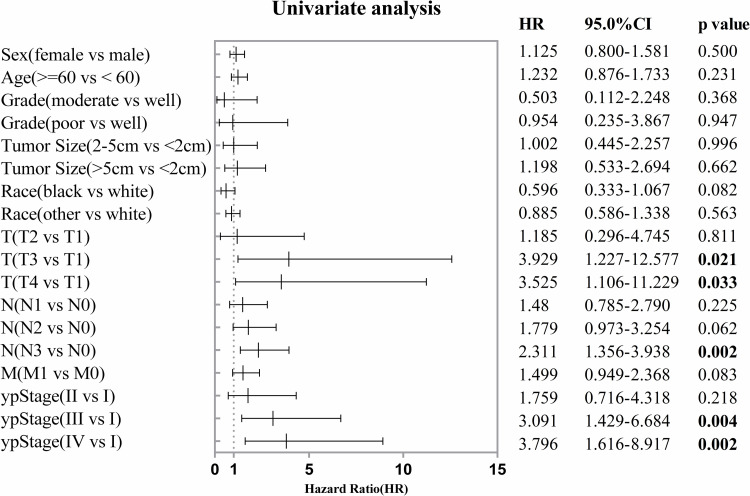
Forest plot displaying univariate Cox regression analysis results of factors related to OS.

**Fig 4:**
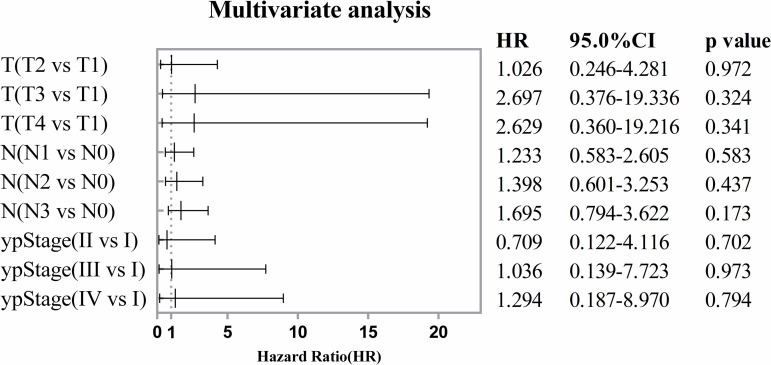
Forest plot displaying multivariate Cox regression analysis results of factors related to OS.

This analysis encompassed a range of common influencing factors related to the prognosis of GC. The univariate analysis demonstrated that T category, N category, and ypStage are factors influencing the overall survival of GC patients following preoperative treatment (Fig 3). Nevertheless, the multivariate Cox regression analysis revealed that T category, N category, and ypStage were not independent factors influencing the overall survival of GC patients undergoing preoperative chemotherapy or chenmradiotherapy (Fig 4).

## Discussion

In recent years, preoperative therapy has become a widely used modality in the treatment of advanced GC. It is imperative for physicians to ascertain the prognosis of patients who have undergone neoadjuvant chemotherapy, in order to make an appropriate clinical decision. The 8th edition of the TNM staging manual proposed the ypTNM system for patients undergoing radical surgery with preoperative therapy, thereby establishing a uniform standard for evaluating patients after preoperative therapy for the first time. The manual presented the one-year, three-year and five-year survival rates, as well as the median survival time, for GC patients with varying ypTNM stages. Patients with GC and a specific ypTNM stage exhibites a poorer prognosis than those with the same stage defined by pTNM [7].

However, the ypTNM staging system still exhibited some shortcomings. For instance, the system was based on data from 683 patients, and its accuracy required validation with larger sample data. The ypTNM staging system indicated the status of tumour infiltration, lymphatic metastasis and distant metastasis after preoperative therapy. However, it was unable to indicate the degree of tumour remission before and after treatment, which is crucial for guiding further treatment and predicting prognosis. Furthermore, the ypTNM staging system categorised non-metastatic GC patients after preoperative therapy into three stages: stage I, stage II and stage III, without making more detailed distinctions. In order to address the shortcomings of the ypTNM staging system, Zhang *et al.* developed and validated a modified ypTNM staging through multicenter data that is superior to the AJCC 8th edition ypTNM staging, allowing more accurate assessment of the prognosis of patients with GC after neoadjuvant therapy [8]. Li *et al.* established a reliable nomogram to predict the one- and three-year OS of patients who received neoadjuvant chemotherapy and radical gastrectomy with D2 lymph node dissection [9].

In this study, we sought to address another question that arises in clinical practice, namely whether the prognosis of GC patients with the same ypStage is consistent after receiving different preoperative therapies. The manual did not address this question. The findings of our research indicated that patients with the same ypStage who received preoperative chemoradiotherapy before surgery exhibites a markedly inferior prognosis. In patients with ypStage III disease, this difference was particularly pronounced. This suggested that the current ypTNM staging system is still imperfect and that it is inappropriate to rely on a single staging system without considering the impact of preoperative treatment.

In comparison to chemotherapy, chemoradiotherapy was associated with higher rates of pathological complete response (pCR) and pathological downstaging. The POET study comprised 119 patients with locally advanced esophagogastric junction (EGJ) tumors and sought to evaluate the comparative efficacy of preoperative chemotherapy and preoperative chemoradiotherapy. The results demonstrated that preoperative chemoradiotherapy markedly elevated the pCR rate (15.6% vs. 2.0%, P = 0.03) and tumor-free lymph node rate (64.4% vs. 36.7%, P = 0.001) in patients [10]. The RTOG9904 multicenter phase II clinical trial administered preoperative chemoradiotherapy to 49 patients with T1-3N0-1 stage gastric cancer (GC). Among the cohort, 77% of patients achieved R0 resection, with a pCR rate of 26% [11]. Tsai et al. included a total of 5,371 non-metastatic EGJ patients in their retrospective analysis. The results demonstrated that a greater proportion of patients who received radiotherapy underwent R0 resection (91.4% vs. 86.6%, P <  0.001) [12]. Chemoradiotherapy can more effectively downstage tumors due to the combined effects of radiation and chemotherapy. Radiotherapy destroys the DNA of tumor cells, inhibiting their growth and division. Additionally, it disrupts the DNA repair mechanisms of tumor cells, making them more susceptible to chemotherapy [13]. However, preoperative chemoradiotherapy has a more pronounced effect on reducing tumor staging, which suggests that patients may have more advanced clinical staging at the time of diagnosis, or that the tumor is resistant to radiotherapy and chemotherapy. This may elucidate why our analysis showed that patients who undergo preoperative chemoradiotherapy exhibit a poorer prognosis under the same ypStage.

The results of the Cox regression analysis demonstrated that ypT, ypN, and ypStage are not independent factors influencing patient OS. This may be attributed to the pathological ypTNM staging system, which provides an assessment of the patient’s tumour status at the time of surgery but does not take into account the tumour regression. The impact of tumour regression grade (TRG) on overall survival in patients undergoing adjuvant therapy has been corroborated by a multitude of studies [14,15]. Fareed *et al.* identified a positive correlation between TRG and ypTNM staging [16]. Additionally, research findings suggested that histopathological response is a significant predictor of prolonged OS in patients with ypN0-1 [17]. It can be surmised that the combination of ypTNM and TRG grading may prove an efficacious method for achieving an accurate prediction.

It should be noted that this study is not without limitations. Firstly, this is a retrospective study originating from the SEER database, which introduces the potential for data loss and selection bias. Secondly, the SEER database does not provide data on the time of recurrence, which precludes an evaluation of the progression-free survival of GC patients. Thirdly, the number of cases included in this study is relatively limited, particularly in the chemoradiotherapy group, which reduces the reliability of the validation. It should be noted that the SEER database contains a considerable number of cases in which patients received preoperative adjuvant chemotherapy or chemoradiotherapy. However, only a subset of patients from 2016 to 2017, for whom postoperative pathological results were available, were included in this study. The use of targeted therapy and immunotherapy is gradually becoming more prevalent in preoperative treatment. However, due to the limited number of cases available for analysis, this study was unable to ascertain whether there are differences in prognosis between patients who have undergone such treatment.

## Conclusion

The prognosis of GC patients in the same ypStage but receiving different preoperative treatments is markedly disparate. It is recommended that the forthcoming iteration of the ypStage system should differentiate between various preoperative treatments, particularly in view of the growing utilisation of preoperative targeted therapy and immunotherapy.
